# Misdiagnosed, mismanaged, mistreated: personality disorders and the Mental Health Act

**DOI:** 10.1192/bjb.2025.10124

**Published:** 2026-06

**Authors:** Ruslan Zinchenko, Hosam Elhamoui

**Affiliations:** 1 Psychotherapy Service, Lancashire and South Cumbria NHS Foundation Trust, Preston, UK; 2 Specialist Psychotherapy Service, Greater Manchester Mental Health NHS Foundation Trust, Manchester, UK

**Keywords:** Personality disorders, Mental Health Act, compulsory detention, medical law and ethics, iatrogenic harm

## Abstract

The Mental Health Act perpetuates the harmful and misguided detention of individuals with personality disorders. The outdated practice lacks ethical, legal or clinical justification. Coercion is mistaken for care, and detention often exacerbates distress, retraumatises patients and increases suicide risk. Despite its promises, the new Mental Health Bill fails to address these systemic failures, continuing the cycle of risk-driven, defensive psychiatry. It is time to abandon compulsory detention for this patient group, redirect resources toward evidence-based, relational interventions, and move toward a capacity-based, trauma-informed legal framework that aligns with contemporary psychiatric understanding of these conditions and fundamental human rights.

The prevailing assumption in psychiatric practice, embedded within the Mental Health Act 1983/2007 (MHA), is that individuals with a personality disorder can be objectively assessed and managed, with compulsory detention criteria applied in a manner similar to other conditions, such as schizophrenia or bipolar disorder.

This article challenges this assumption, arguing that the fundamental relational nature of personality disorders renders traditional assessment frameworks under Part 2 of the MHA not only inadequate, but potentially harmful.

Furthermore, considering the restrictive and controlling nature of in-patient psychiatric care, and the lack of overall therapeutic approach and evidence-based interventions on in-patient units, the legal and ethical justification for compulsory detention of individuals with personality disorders is unclear. Despite the intended progressive reforms of the Mental Health Bill (MHB), its capacity to change the entrenched patterns of mismanagement in this patient group is questionable.

## Rethinking personality disorders

Individual personality refers to enduring patterns of thoughts, emotions, motivations and behaviours that shape an individual’s way of relating to themselves and others. Although personality is inherently adaptive, personality disorders are characterised by rigid and maladaptive patterns of interpersonal functioning, causing significant impairment. In practice, diagnosing personality disorders is challenging because of their variability, symptom instability and overlap with normal personality traits and other psychiatric conditions.

Historically, in line with the medical disease model, personality disorders were conceptualised categorically, but contemporary research highlights their dimensional nature. Rather than fixed, discrete conditions, personality disorders are now understood to exist along a spectrum, with significant variability influenced by context and relationships.

Personality disorders have strong developmental underpinnings, with early-life adversity strongly predicting the severity. Attachment theory posits that early caregiving interactions shape internal relational models, influencing future interpersonal functioning. Secure attachments promote trust and stability, whereas adverse childhood experiences lead to maladaptive relational templates and epistemic mistrust. Mentalisation theory explains how disorganised attachment disrupts reflective functioning undermining relational stability.

Interpersonal theory conceptualises personality pathology as inherently relational, arising from difficulties in how individuals perceive, interact with, and understand themselves in relation to others. From this perspective, personality disorders differ from other psychiatric conditions because their primary manifestation is in relationships, and could therefore be better thought of as interpersonal disorders.

## Trapped by the MHA

MHA assessments are inherently relational encounters that are shaped by unconscious relational dynamics that may distort decision-making. These assessments often involve power imbalances that reinforce dysfunctional relational patterns. Assessors often find themselves in a double-bind, not because of any failing on their part or the patient’s, but because of the very nature of the personality difficulty itself. As a result, their decision can end up reflecting the patient’s unconscious dynamics, either through detention, which echoes past experiences of coercion and control, or discharge, which may feel like rejection or abandonment. In both cases, there is a risk of unintentionally re-enacting earlier trauma.

Individuals with a personality disorder often present to emergency services in crisis, leading to repeated detentions related to ‘risk management’. The trauma of repeated crisis detentions, particularly those involving police, can further contribute to relational difficulties and mistrust of services, ultimately increasing recurrent suicidal crises.^1^ This is concerning, as frequent psychiatric emergency presentations have been identified as a strong predictor of suicide attempts, with each additional visit further increasing the risk.^2^

## Detained but not treated

Psychiatrists frequently detain individuals with personality disorders under the MHA despite the lack of evidence-based interventions delivered in a compulsory in-patient setting.^3^

Paradoxically, contrary to its therapeutic intention, compulsory in-patient admissions for individuals with personality disorders frequently result in developmental regression, worsening psychopathology and heightened suicidal behaviour.^4^ Involuntary psychiatric detention is frequently experienced as distressing, coercive and re-traumatising; patients often report fear, powerlessness and a lack of autonomy, and perceive coercive measures such as restraint and forced medication as punitive.^5^

Some aspects of hospital admission itself, such as trauma, loss of autonomy and social disruption, may contribute to suicidality. Patients with personality disorders may be particularly vulnerable to these experiences because admissions often exacerbate their distress. A dose−response relationship has been observed, with longer or repeated admissions correlating with higher suicide rates, raising concerns that hospital admission may not always be protective.^6^

Furthermore, locked in-patient environments do not necessarily reduce risk, and evidence suggests that risk can be effectively managed in open residential settings, such as therapeutic communities, which prioritise relational security, structured support and patient autonomy over coercion and restriction.^7^

## When law and ethics collide

One of the more controversial aspects of compulsory detention of individuals with a personality disorder is the questionable ethical justification, given that many patients retain decision-making capacity even during crisis episodes.^8^ The justification for overriding autonomy based purely on perceived risk is increasingly untenable, as it conflicts with broader principles in medical ethics and law, where competent patients’ decisions, however unwise, are respected.

Comparatively, under the MHA, individuals cannot be detained solely for substance addiction, recognising their right to make unwise decisions. Despite addiction undoubtedly being a disturbance of the mind, involving neurocognitive adaptations that drive harmful decision-making, this standard is not applied to personality disorders, highlighting legislative inconsistency.

Additionally, clinicians often admit that risk-driven compulsory detentions are influenced by defensive practices motivated by fear of litigation, media scrutiny or personal anxiety rather than genuine clinical benefit.^9^ Furthermore, it is ethically and legally problematic to justify compulsory detention solely based on suicide risk assessments, which remain fundamentally unreliable and imprecise.^10^

It is also worth noting that some suicides may occur despite good psychiatric care, just as myocardial infarctions still occur despite good cardiac care. The pursuit of total risk elimination through coercive measures may paradoxically worsen outcomes. As a profession, psychiatrists must take a leading role in helping wider society, policy makers and legislators come to terms with the limits of our interventions.

## The Mental Health Bill: progress or more of the same?

The new MHB introduces four principles intended to guide mental health practice toward more ethical and patient-centred care. However, for individuals with a personality disorder, these principles are unlikely to translate into meaningful change without specific legislative provisions.

The principle of choice and autonomy is frequently compromised in practice, as these patients commonly retain the capacity to make decisions, albeit unwise, even in crisis. Detaining someone who understands the implications of their decisions undermines autonomy and contravenes general medical ethical and legal principles. In medicine, competent patients can refuse life-saving treatment, yet this standard is inconsistently applied in psychiatry.

The principle of least restriction is similarly challenged by the routine application of overly restrictive measures, including restraints and compulsory medication. These often re-traumatise the patient and result in self-perpetuating cycles of behavioural disturbance and restraint.

The principle of therapeutic benefit is particularly problematic given that evidence-based therapies are scarcely delivered in in-patient units, and no psychotropic medication has been licensed or shown to be effective. Thus, detaining these patients frequently lacks genuine therapeutic justification, raising questions about its legality and ethical validity.

Finally, the principle of seeing the person as an individual is undermined by the coercive nature of compulsory detentions, which are often risk-focused and result in further fragmentation of an already unstable identity.

## Beyond coercion, toward meaningful reform

The new MHB, although well-intentioned, fails to sufficiently address the systemic and conceptual flaws in how patients with personality disorders are assessed and treated under the MHA. For genuine change, legislation must explicitly recognise the fundamentally relational and interpersonal nature of personality disorders. It must move away from risk-focused, restrictive and coercive care, and toward a more capacity-based, trauma-informed and relational framework in line with contemporary understanding of these conditions.

The MHB should actively limit compulsory in-patient detention, especially when patients are competent to make decisions, even if those are perceived as unwise by others. One way would be fusion legislation, exemplified by Northern Ireland’s Mental Capacity Act (2016), which merges mental health and capacity laws. Under such a framework, individuals with decision-making capacity cannot be involuntarily detained. It is important to recognise that apparently unwise decision-making in an individual with a personality disorder is contextual, and often represents an interpersonal phenomena rather than an internal temporary but remediable state. As such, the process of capacity assessment could not be applied in its current form without relevant modifications.

It is difficult to justify compulsory detention based on risk when detention itself increases risk. Although brief informal crisis admissions to structured, personality disorder-informed therapeutic settings may be beneficial, repeated detentions under the MHA Section 2 for individuals with well-established diagnoses add little clinical value and should be restricted legally.

For example, a modification to the MHA criteria for admission under Section 2 should actively restrict detention of patients whose presentation is consistent with their diagnosis and known difficulties. A clause explicitly stating that the detention in a hospital setting is unlikely to worsen the patient’s mental disorder could also be added.

Detentions under MHA Section 3 should be abolished, as evidence-based treatments for personality disorders cannot be delivered under coercion. We propose that in complex cases, where MHA Section 3 is currently used, consideration should be given to informal long-term residential units. Until legal coercion is available and widely used, such units are unlikely to be adopted and developed.

The essence of the proposal in this article is rooted in the principle of ‘do no harm’ and would align mental health law with ethical principles and contemporary scientific understanding.

## About the authors

Ruslan **Zinchenko** is a specialty registrar (ST7) in general psychiatry and medical psychotherapy in a psychotherapy service in Lancashire and South Cumbria NHS Foundation Trust, Preston, UK. **Hosam Elhamoui** is a consultant in medical psychotherapy and general psychiatry in a specialist psychotherapy service, in Greater Manchester Mental Health NHS Foundation Trust, Manchester, UK.
